# Dietary inflammatory index could increase the risk of sarcopenia in patients with chronic kidney disease

**DOI:** 10.1038/s41598-024-65340-6

**Published:** 2024-07-03

**Authors:** Fatemeh Mansouri, Fatemeh Jafari, Sara Ranjbar, Fateme Souni, Shahrokh Ezzatzadegan Jahromi, Zainab Shateri, Siavash Babajafari, Mehran Nouri

**Affiliations:** 1 Department of Clinical Nutrition, School of Nutrition and Food Sciences, Shiraz University of Medical Sciences, Shiraz, Iran; 2 Department of Nutritional Sciences, Chandlee Lab, Penn State University, University Park, PA 16802 USA; 3 Obesity and Metabolic Health Laboratory, Department of Nutritional Sciences, College of Human Sciences, Texas Tech University, Lubbock, TX 79409 USA; 4 Student Research Committee, Shiraz University of Medical Sciences, Shiraz, Iran; 5 Department of Medicine, School of Medicine, Shiraz Nephro-Urology Research Center, Shiraz University of Medical Sciences, Shiraz, Iran; 6 Department of Nutrition and Biochemistry, School of Medicine, Ilam University of Medical Sciences, Ilam, Iran; 7 Nutrition Research Center, Department of Clinical Nutrition, School of Nutrition and Food Sciences, Shiraz University of Medical Sciences, Shiraz, Iran; 8 Mobility Impairment Research Center, Health Research Institute, Babol University of Medical Sciences, Babol, Iran; 9 Health Policy Research Center, Institute of Health, Shiraz University of Medical Sciences, Shiraz, Iran

**Keywords:** Dietary inflammatory index, Protein–energy wasting, Sarcopenia, Chronic kidney disease, Health care, Medical research, Nephrology, Risk factors

## Abstract

Using a system that incorporates a variety of food items rather than focusing on individual components can aid in assessing the inflammatory effects of a diet on disease outcomes such as chronic kidney disease (CKD). Therefore, we decided to investigate the association between dietary inflammatory index (DII) and the risk of protein–energy wasting (PEW) and sarcopenia in patients with CKD. In this cross-sectional study, 109 patients with CKD were selected from two clinics in Shiraz, Iran. The intake of individuals’ diets was recorded using a validated 168-item food frequency questionnaire. Additionally, Asian Working Group for Sarcopenia (AWGS) guidelines were utilized to evaluate muscles’ strength, mass, and function. Also, four International Society of Renal Nutrition and Metabolism (ISRNM) criteria (body mass index, intake of protein, albumin, and urine creatinine) were used  to diagnose PEW. Logistic regression was used to assess the association between DII and sarcopenia as well as PEW. The results showed that the intake of saturated fatty acids, trans fatty acids, niacin, beta-carotene, and vitamin C was significantly different between lower and higher DII groups. In the univariate model, higher odds of sarcopenia was observed by each unit increase in DII (odds ratio (OR) = 1.379, 95% confidence interval (CI): 1.042–1.824) and age (OR = 1.073, 95% CI: 1.017–1.132). Additionally, in the multivariate model, the association between DII and age with odds of sarcopenia remained significant (DII: OR = 1.379, 95% CI: 1.030–1.846 and age: OR = 1.063, 95% CI: 1.007–1.121). The current study suggests the possible role of pro-inflammatory foods in worsening muscle health, specifically sarcopenia, in CKD patients. Future longitudinal studies may reveal the causative nature of these correlations.

## Introduction

Chronic kidney disease (CKD) is defined as the progressive loss of kidney function leading to irreversible kidney failure^1^. The global prevalence of CKD in 2022 was estimated to be more than 10%^2^; however, studies have shown a higher prevalence of CKD among the Iranian population^3^.

Loss of muscle mass and function, known as sarcopenia, is a frequent finding in CKD, particularly in end-stage renal disease (ESRD)^4^. Negative protein balance can result from various factors such as anorexia, increased energy expenditure, metabolic acidosis, insulin resistance, hormonal degradation, and inflammation^5^. Loss of muscle mass, especially skeletal muscle, is associated with lower quality of life, cardiovascular complications, graft loss, and postoperative complications in kidney transplant, higher risk of falls, loss of personal autonomy, hospitalization and death^6–8^. Therefore, sarcopenia is considered a negative prognostic factor in CKD patients and requires prevention and treatment in these patients.

Also, protein–energy wasting (PEW), a state of nutritional and metabolic imbalances, is characterized by the loss of body protein and energy stores, leading to muscle and fat mass loss and cachexia in CKD patients. PEW can be caused by hypermetabolic status, uremic toxins, malnutrition, and inflammation^9^.

Previous interventions to prevent and treat sarcopenia have focused on increasing protein intake^10^. However, inflammation is considered one of the underlying mechanisms explaining sarcopenia. Studies have demonstrated that low-grade inflammation may contribute to the development of sarcopenia^11,12^. For instance, a systematic review and meta-analysis showed that inflammatory cytokines are inversely associated with muscle strength and mass^12^. CKD progression is known to lead to inflammation and oxidative stress^13^. Indeed, individuals with CKD typically experience low-grade chronic inflammation^14^.

Previous studies have shown that various food items such as whole grains, fruits, vegetables, fish, and certain nutrients including vitamins E and C, omega-3, and magnesium have anti-inflammatory properties. Conversely, refined sugars, simple sugars, red meat, and high-fat dairy have been shown to have pro-inflammatory effects^15,16^. Using a system that incorporates a variety of food items rather than focusing on individual components can aid in assessing the inflammatory effects of a diet on disease outcomes such as CKD.

The dietary inflammatory index (DII) is a literature-based scoring system designed to evaluate the inflammatory potential of a diet by considering both pro- and anti-inflammatory food items^17^. Previous studies have shown a negative association between DII and various diseases such as colorectal cancer^18^, prediabetes and insulin resistance^19^, frailty, and 8-year mortality^20^, as well as the risk and progression of kidney disease^21,22^. A meta-analysis study indicated an adverse association between DII and sarcopenia^23^. However, there is limited evidence regarding the association between DII and sarcopenia in individuals with CKD in the Iranian population. To our knowledge, only one study based on the National Health and Nutrition Examination Survey (NHANES) has shown a positive association between higher DII and sarcopenia in CKD patients^24^. Giving the different dietary habits between the United States (US) and Iran, our study aimed to investigate the association between DII and the risk of PEW and sarcopenia in patients with CKD.

## Methods

### Study population

This cross-sectional study was conducted at Motahari and Imam Reza clinics in Shiraz, Fars province, Iran, between January and October 2022. Some books or guidelines suggest using an expected prevalence of 50% if we cannot obtain the prevalence at all^25–27^, and the sample size was calculated based on this assumption. The sample size was calculated as 97 individuals (with (p) = 0.5, d = 0.1, and α = 0.05), and accounting for a 12% drop-out rate, the estimated number of participants was 109 individuals.

The inclusion criteria were age > 18 years, glomerular filtration rate (GFR) less than 60 mL/min/173m^2^, and the absence of cognitive problems. Conversely, the exclusion criteria were an energy intake of more than 4200 or less than 800 kcal/day and a questionnaire response rate less than 60%. This study was approved by the Medical Research and Ethics Committee of Shiraz University of Medical Sciences (IR.SUMS.SCHEANUT.REC.1402.061) and conducted in accordance with the ethical standards of the Declaration of Helsinki.

### Data collection

The percentage of muscle and fat mass was assessed by bioelectrical impedance analysis (BIA). Participants’ weight was measured with a precision of 0.1 kg, and the height and mid-arm circumference (MAC) were determined with an accuracy of 0.5 cm. Also, body mass index (BMI) was calculated. Physical activity was assessed using the International Physical Activity Questionnaire (IPAQ)^28^. For evaluation of biochemical variables, 5 mL blood samples were collected from each participants.

### Sarcopenia and PEW diagnosis

The Asian Working Group for Sarcopenia (AWGS) guidelines were utilized to evaluate muscle strength, mass, and function^29,30^. Handgrip strength (HGS) was measured using a dynamometer to assess muscle strength. Muscle function was evaluated using gait speed (walking speed in 6 m) and the five-time chair-stand test. Skeletal muscle mass index (SMI) was calculated as skeletal muscle mass divided by the square of height. The criteria for the diagnosis of sarcopenia were as follows: SMI < 7 kg/m^2^ for men or SMI < 5.7 kg/m^2^ for women was the first criterion. Additionally, a gait speed of fewer than 1 m/s or more than 12 s in the five-time chair-stand test, and/or an HGS of less than 28 kg for men and 18 kg for women, were used to confirm the diagnosis of sarcopenia^29,30^.

According to the International Society of Renal Nutrition and Metabolism (ISRNM), four criteria were used to distinguish PEW: BMI < 23 kg/m^2^, protein intake  < 0.6 g/kg/day, biochemical parameters such as albumin < 3.8 g/dL, and lower quartile of urine creatinine excretion (UCE) in 24 h based on sex. PEW was defined as meeting three or more of these criteria^31^.

### Dietary assessment

The intake of individuals’ diets was recorded using a validated 168-item food frequency questionnaire (FFQ)^32^. After calculating food grams and multiplying by the daily intake frequency, the amount of each item was determined. FFQ was used to calculate the DII. Initially, the residual method was employed to adjust energy intake for each nutrient^33^. The DII score was determined according to the method described by Shivappa et al.^34^. To calculate DII, 31 food parameters were considered, including energy, fat, protein, carbohydrates, various vitamins (B_1_, B_2_, B_3_, B_6_, B_9_, B_12_, C, A, D, and E), beta-carotene, fiber, trans fatty acid (TFA), cholesterol, saturated fatty acid (SFA), polyunsaturated fatty acids (PUFA), monounsaturated fatty acids (MUFA), iron, selenium, magnesium, zinc, omega-6, omega-3, caffeine, onion, garlic, and tea.

For each participant, a Z-score was calculated by subtracting the mean of the global standard from the amount consumed and dividing it by the global standard deviation (SD). This Z-score was then converted to a percentile, multiplied by two, and adjusted by subtracting one, to minimize the influence of outliers. Additionally, this value was multiplied by the corresponding food inflammatory effect score to obtain the specific DII score for each food parameter. Finally, the overall DII score was calculated by summing all these individual scores. A higher DII score indicates a higher pro-inflammatory potential of the diet.

### Statistical analysis

For statistical analysis, SPSS software (version 22) was used. Continuous variables were described using mean ± SD or median (interquartile range (IQR)), while categorical variables were presented as percentages. An independent sample T-test assessed parametric continuous variables, whereas the Mann–Whitney test was employed for non-parametric continuous variables. The chi-square test was applied for categorical variables. Additionally, logistic regression was conducted to assess the association between DII and sarcopenia as well as PEW. The multivariate model was adjusted for age, energy, protein, fat, and salt intake, sex and smoking history. Statistical significance was defined as a p-value less than 0.05.

### Ethics approval and consent to participate

This study was approved by the medical research and ethics committee of Shiraz University of Medical Science and the informed consents were completed by all participants.

## Results

The baseline features of the study population, according to the lower and higher mean of DII, are reported in Table 1. The median total iron-binding capacity (TIBC) (*P* = 0.031) and the DII total score (P˂0.001) were significantly greater in the higher DII group compared to the lower DII group. Other variables did not show significant differences between the two groups.

**Table 1:** Baseline characteristics of the study participants across the median of DII.

Variables	Dietary inflammation index		
	Lower than mean (*n* = 56)	Higher than mean (*n* = 53)	P-value
Age (year) ^1^	64.5 (15.0)	60.5 (26.0)	0.215
BMI (kg/m^2^) ^2^	28.66 ± 5.37	27.36 ± 5.92	0.227
Walk duration (second) ^1^	7.0 (1.5)	6.0 (2.0)	0.312
Chair sitting (second) ^1^	14.0 (2.3)	13.0 (4.1)	0.248
Muscle weight (kg) ^1^	21.93 ± 5.29	21.64 ± 5.63	0.783
Fat percentage (%) ^2^	28.41 ± 9.53	25.90 ± 9.55	0.171
MAC (cm) ^1^	29.0 (3.0)	29.0 (5.0)	0.817
ASM (kg/m^2^) ^2^	8.37 ± 1.32	8.22 ± 1.75	0.616
HGS (kg) ^1^	20.0 (15.0)	16.5 (13.0)	0.945
Sarcopenia, yes (%) ^3^	5 (8.8)	11 (20.8)	0.106
PEW, yes (%) ^3^	7 (12.3)	9 (17.0)	0.592
Smoking, yes (%) ^3^	15 (26.3)	8 (15.1)	0.167
Sex, male (%) ^3^	29 (50.9)	31 (58.5)	0.449
SGA ^1^	8.0 (3.0)	9.0 (5.0)	0.349
Physical activity (%) ^3^			0.404
Low	43 (75.4)	36 (67.9)	
Moderate	14 (24.6)	17 (32.1)	
PTH (pg/mL) ^1^	67.5 (53.4)	57.0 (80.0)	0.084
Vitamin D_3_ level (ng/mL) ^2^	34.03 ± 14.44	35.49 ± 16.24	0.625
GFR (mL/min/1.7m^2^) ^2^	31.07 ± 13.85	34.36 ± 13.01	0.202
Hemoglobin (gr/dL) ^2^	12.57 ± 2.08	12.50 ± 2.23	0.872
Albumin (gr/dL) ^2^	4.04 ± 0.45	4.13 ± 0.39	0.268
Iron (µg/dL) ^2^	71.24 ± 33.67	73.95 ± 35.93	0.725
Ferritin (ng/mL) ^2^	121.64 ± 83.07	129.63 ± 157.50	0.754
TIBC (mcg/dL) ^1^	279.0 (89)	316.5 (62.0)	**0.031**
BUN (mg/dL) ^2^	33.66 ± 17.64	30.21 ± 12.35	0.236
Creatinine (mg/dL) ^1^	1.99 (1.02)	1.96 (1.04)	0.303
Urine creatinine (mg/dL) ^2^	891.74 ± 402.45	847.33 ± 385.96	0.557
FBS (mg/dL) ^1^	99.5 (47.0)	96.0 (19.0)	0.365
TG (mg/dL) ^2^	139.02 ± 78.87	163.29 ± 99.24	0.194
Total cholesterol (mg/dL) ^2^	150.65 ± 40.77	153.17 ± 36.74	0.735
LDL-C (mg/dL) ^2^	86.96 ± 30.55	81.33 ± 22.06	0.326
HDL-C (mg/dL) ^2^	43.62 ± 11.66	41.80 ± 9.32	0.507
PCO_2_ (mmHg) ^2^	46.58 ± 52.07	41.80 ± 9.32	0.519
PO_2_ (mmHg) ^1^	32.6 (18.1)	29.8 (16.9)	0.776
HCO_3_ (mmol/L) ^2^	21.96 ± 3.60	23.22 ± 3.47	0.065
DII ^2^	− 1.58 ± 1.60	1.60 ± 1.18	**˂0.001**

BMI: body mass index, MAC: mid-arm circumference, ASM: appendicular skeletal muscle mass, HGS: handgrip strength, PEW: protein–energy wasting, SGA: subjective global assessment, ALT: alanine transaminase, AST: aspartate transaminase, PTH: parathyroid hormone, GFR: glomerular filtration rate, TIBC: total iron-binding capacity, BUN: blood urea nitrogen, FBS: fasting blood sugar, TG: triglyceride, LDL-C: low-density lipoprotein cholesterol, HDL-C: high-density lipoprotein cholesterol, PCO2: partial pressure of carbon dioxide, PO_2_: partial pressure of oxygen, DII: dietary inflammatory index, M: median.

Values are mean ± SD or median (IQR) for continuous and percentage for categorical variables.

^1^Using Mann–Whitney U test for non-parametric variables.

^2^Using independent sample T-test for parametric variables.

^3^Using chi-square test for categorical variables.

Significant values are in bold.

The intake of DII components based on lower and higher than the mean DII is shown in Table 2. According to the table, SFA (*P* = 0.001), TFA (*P* = 0.017), niacin (*P* = 0.036), beta-carotene (*P* = 0.005), and vitamin C (*P* = 0.033) were significantly different between the two groups.

**Table 2:** Intake of DII components across the median of DII.

Variables	Dietary inflammation index		*P*-value
	Lower than mean *(n* = 57)	Higher than mean (*n* = 53)	
Energy (kcal) ^1^	1786.62 (571.05)	2072.55 (815.68)	**0.035**
Carbohydrate (gr) ^1^	300.68 (115.79)	335.31 (150.66)	0.072
Total Fiber (gr) ^2^	21.73 ± 4.76	21.17 ± 6.53	0.607
Total Lipid (gr) ^1^	47.50 (15.57)	49.28 (26.99)	0.068
Cholesterol (mg) ^1^	128.02 (95.14)	149.34 (128.37)	0.404
SFA (gr) ^1^	12.92 (8.74)	16.99 (13.18)	**0.001**
Trans Fat (gr) ^1^	0.10 (0.18)	0.19 (0.30)	**0.017**
MUFA (gr) ^1^	16.93 (6.39)	17.64 (12.12)	0.431
PUFA (gr) ^1^	11.18 (4.08)	10.57 (4.89)	0.753
Omega-3 (gr) ^1^	0.11 (0.07)	0.11 (0.08)	0.691
Omega-6 (gr) ^1^	7.88 (3.77)	7.30 (5.09)	0.921
Protein Intake (gr) ^1^	52.31 (16.95)	59.40 (27.08)	0.139
Thiamin (mg) ^1^	0.92 (0.37)	0.97 (0.48)	0.394
Riboflavin (mg) ^2^	1.11 ± 0.29	1.16 ± 0.42	0.491
Niacin (mg) ^1^	32.16 (17.37)	38.17 (26.46)	**0.036**
Pyridoxine (mg) ^2^	1.42 ± 0.31	1.49 ± 0.44	0.340
Folate (mcg) ^2^	200.11 ± 46.77	183.52 ± 65.49	0.127
Vitamin B_12_ (mcg) ^1^	1.89 (1.15)	2.08 (1.54)	0.226
Beta-carotene (mcg) ^1^	4812.83 (2062.49)	3817.74 (2182.15)	**0.005**
Vitamin A (RE) ^1^	545.69 (286.21)	491.19 (266.38)	0.073
Vitamin C (mg) ^1^	131.83 (44.66)	117.73 (54.04)	**0.033**
Vitamin D (mcg) ^1^	19.42 (16.13)	18.78 (17.71)	0.983
Vitamin E (mg) ^1^	4.84 (2.21)	4.44 (3.35)	0.605
Magnesium (mg) ^2^	266.39 ± 65.94	283.77 ± 87.76	0.241
Iron (mg) ^1^	10.10 (3.23)	10.76 (5.13)	0.265
Selenium (mcg) ^1^	36.90 (13.54)	41.43 (23.17)	0.240
Zinc (mg) ^1^	8.68 (2.67)	9.90 (4.96)	0.047
Caffeine (mg) ^1^	25.95 (51.92)	51.91 (32.01)	0.993
Garlic (gr) ^1^	1.20 (2.75)	1.20 (1.52)	0.194
Onion (gr) ^1^	33.66 (22.00)	33.66 (15.50)	0.167
Tea (cc) ^1^	236.00 (472.0)	472.00 (291.07)	0.993

DII: dietary inflammatory index, SFA: saturated fatty acid, MUFA: monounsaturated fatty acid, PUFA: polyunsaturated fatty acid, RE: retinol equivalent, M: median, kcal: kilocalorie, gr: gram, mg: milligram, mcg: microgram.

Values are mean ± SD or median (IQR) for continuous variables.

^1^ Using Mann–Whitney U test for non-parametric variables.

^2^ Using independent sample T-test for parametric variables.

Significant values are in bold.

The association between the DII score and the odds of sarcopenia in univariate and multivariate models is shown in Table 3. In the univariate model, a significant increase in the odds of sarcopenia was observed with each unit increase in DII (odds ratio (OR) = 1.379, 95% confidence interval (CI): 1.042–1.824) and age (OR = 1.073, 95% CI: 1.017–1.132). In the multivariate model, the association between DII and age with the odds of sarcopenia remained significant (DII: OR = 1.379, 95% CI: 1.030–1.846 and age: OR = 1.063, 95% CI: 1.007–1.121).

**Table 3:** Association between the dietary inflammatory index and the odds of sarcopenia in two uni- and multivariate models.

Variables	Univariate		Multivariate	
	OR (CI 95%)	*P*-value	OR (CI 95%)	*P*-value
DII	**1.379 (1.042–1.824)**	**0.024**	**1.379 (1.030–1.846)**	**0.031**
Age (year)	**1.073 (1.017–1.132)**	**0.009**	**1.063 (1.007–1.121)**	**0.027**
Energy intake (kcal/day)	1.000 (0.999–1.001)	0.959	1.000 (0.997–1.003)	0.960
Protein intake (g/day)	1.001 (0.972–1.031)	0.945	1.009 (0.925–1.003)	0.838
Fat intake (g/day)	0.990 (0.959–1.022)	0.535	0.980 (934–1.029)	0.418
Salt intake (g/day)	1.119 (0.582–2.153)	0.736	1.014 (0.501–2.431)	0.807
Sex				
Female	Ref	Ref	Ref	Ref
Male	1.467 (0.493–4.363)	0.491	1.545 (0.451–5.293)	0.489
Smoking history				
No	Ref	Ref	Ref	Ref
Yes	0.854 (0.222–3.291)	0.818	0.813 (0.182–3.620)	0.786

OR, odds ratio; CI, confident interval; DII, dietary inflammatory index; Ref, reference.

Obtained from logistic regression and using enter method for multivariate analysis.

These values are odds ratios (95% CIs).

Significant values are shown in bold.

The association between the DII score and the odds of PEW in univariate and multivariate models is shown in Table 4. In both models, no significant association was observed.

**Table 4:** Association between the dietary inflammatory index and the odds of protein–energy wasting in two uni- and multivariate models.

Variables	Univariate		Multivariate	
	OR (CI 95%)	*P*-value	OR (CI 95%)	*P*-value
DII	1.083 (0.841–1.394)	0.538	1.091 (0.845–1.409)	0.505
Age (year)	1.012 (0.973–1.052)	0.561	1.016 (0.974–1.060)	0.457
Energy intake (kcal/day)	1.000 (0.999–1.001)	0.489	0.998 (0.996–1.000)	0.053
Fat intake (g/day)	1.008 (0.983–1.035)	0.528	1.049 (0.997–1.104)	0.063
Salt intake (g/day)	1.024 (0.534–1.966)	0.943	0.977 (0.476–2.006)	0.950
Sex				
Female	Ref	Ref	Ref	Ref
Male	1.084 (0.373–3.154)	0.882	0.880 (0.280–2.766)	0.827
Smoking history				
No	Ref	Ref	Ref	Ref
Yes	0.497 (0.104–2.361)	0.379	0.493 (0.100–2.435)	0.386

OR, odds ratio; CI, confident interval; DII, dietary inflammatory index; Ref, reference.

Obtained from logistic regression and using enter method for multivariate analysis.

These values are odds ratios (95% CIs).

Significant values are shown in bold.

## Discussion

The main finding of our study was the significant association between a higher DII score and an increased risk of sarcopenia in CKD patients. Dietary inflammation appears to compromise muscle health in these patients. Contrary to expectations, our results did not show a significant association between DII scores and PEW.

Inflammation is common in patients with CKD, and the level of inflammation increases as kidney function worsens. Impaired kidney function results in the accumulation of uremic toxins, which exacerbate inflammatory states^35^. Elevated inflammatory markers, including C-reactive protein (CRP), interleukin (IL)-6, and tumor necrosis factor-alpha (TNF-α), exacerbate muscle protein catabolism, contributing to sarcopenia^36^. Anti-inflammatory interventions have shown promise in reducing CKD progression and attenuating sarcopenia^37,38^. These approaches attempt to disrupt the harmful loop that connects CKD and sarcopenia by targeting inflammation and providing a potential therapeutic tool to enhance the quality of life for CKD patients. Our findings revealed that a lower DII score was associated with less sarcopenia. This finding aligns with previous studies that have reported a positive association between DII and muscle mass in children, as well as sarcopenia in patients with CKD and Crohn’s disease^39–41^.

A pro-inflammatory diet may cause muscle mass loss by intensifying systemic inflammation^12^, and protective dietary patterns should be considered as a modifiable preventive factor for CKD-related sarcopenia. The DII, a novel dietary index that determines the total inflammatory potential of a diet, has been validated against systemic inflammatory biomarkers^42^. The DII score considers how various foods and nutrients interact with each other, rather than examining each nutrient or food independently concerning the disease. Consequently, it may offer more promising methods for disease prevention and control^43,44^.

There are several potential mechanisms explaining how high-inflammatory diets can exacerbate sarcopenia. A pro-inflammatory diet may heighten systemic inflammation, resulting in elevated levels of cytokines that inhibit muscle protein synthesis^40^. This can harm muscular health and increase oxidative stress, insulin resistance, and nutrient deficiencies^45,46^. Additionally, a pro-inflammatory diet may affect the gut flora, leading to muscle wasting by impairing the absorption of nutrients essential for muscle health and increasing systemic inflammation and insulin resistance^47^.

The present study did not show a significant association between DII and PEW. Few studies have investigated this association in CKD patients. However, a cross-sectional study of 105 subjects with ESRD did show associations between PEW and a pro-inflammatory diet^48^. One explanation for this discrepancy is that participants in our study were in the moderate stage of CKD, while the mentioned study examined ESRD. The prevalence of PEW in stages 3–5 is 11–46%, whereas in dialysis patients, it ranges from 28 to 80%^49^. The results may have also been impacted by methodological considerations such as sample size, PEW measurement techniques, or other confounding variables. It is also conceivable that, despite certain areas of overlap, the mechanisms behind sarcopenia and PEW have significant differences^50,51^, and DII might affect only a subset of these pathways. Further research is required to understand the underlying causes of this unequal connection and to consider other potential confounding factors.

In the present study, we observed that levels of SFA and TFA increased, and the levels of niacin, beta-carotene, and vitamin C decreased with an increase in the DII score. Additionally, the DII score was positively associated with sarcopenia status. These components have previously been linked to inflammatory responses in CKD patients. For example, high intakes of SFA and TFA are associated with pro-inflammatory markers and muscle mass^52,53^. Conversely, nutrients such as beta-carotene and vitamin C possess anti-inflammatory and muscle-building properties^54–56^. Although these nutrients were consumed around the recommended dietary allowance (RDA), research has indicated that patients experienced better outcomes when supplemented with nutrients^56,57^. However, more studies are needed to evaluate the safety and effectiveness of combined supplementation of anti-inflammatory nutrients. Furthermore, diets that replace SFA and TFA with healthier MUFA and PUFA have been shown to reduce inflammation levels^52,58^ and have positive effects on animal muscles. However, clinical trials have yielded contradictory results regarding their impact on muscles, necessitating further research^52^.

Although the current study provides insightful findings, it is important to acknowledge potential limitations. Due to the cross-sectional nature of our study, longitudinal studies are still needed to confirm causation and evaluate the long-term effects of diet modification in CKD patients. Also, further research is necessary to elucidate mechanistic pathways linking dietary inflammation to CKD outcomes. Such studies could inform the development of nutritional therapies tailored specifically to the requirements of CKD patients.

Another limitation of the present study was the small sample size. Furthermore, nutrient intake was assessed using the FFQ, which may introduce recall bias. Moreover, certain confounding variables were not controlled for in our study.

## Conclusions

The current study highlighted the possible role of pro-inflammatory foods in exacerbating muscle health issues, specifically sarcopenia, in CKD patients. Future longitudinal studies could elucidate the causal relationships of these associations and potentially facilitate the development of nutritional therapies aimed at mitigating muscle-related problems in CKD.

## Data Availability

The datasets used and/or analyzed during the current study are available from the corresponding author on reasonable request.
